# Fucoidan Protects Dopaminergic Neurons by Enhancing the Mitochondrial Function in a Rotenone-induced Rat Model of Parkinson’s Disease

**DOI:** 10.14336/AD.2017.0831

**Published:** 2018-08-01

**Authors:** Li Zhang, Junwei Hao, Yan Zheng, Ruijun Su, Yajin Liao, Xiaoli Gong, Limin Liu, Xiaomin Wang

**Affiliations:** ^1^Department of Neurobiology,; ^2^Department of Physiology,; ^3^Key Laboratory for Neurodegenerative Disorders of the Ministry of Education, Capital Medical University, Beijing 100069, China.; ^4^The Brain Science Center, Beijing Institute of Basic Medical Sciences, Beijing 100039, China.; ^5^ Beijing Institute for Brain Disorders, Beijing 100069, China

**Keywords:** fucoidan, Parkinson’s disease, mitochondria, rotenone, PGC-1α, NRF2

## Abstract

The mitochondrion is susceptible to neurodegenerative disorders such as Parkinson’s disease (PD). Mitochondrial dysfunction has been considered to play an important role in the dopaminergic degeneration in PD. However, there are no effective drugs to protect mitochondria from dysfunction during the disease development. In the present study, fucoidan, a sulfated polysaccharide derived from *Laminaria japonica*, was investigated and characterized for its protective effect on the dopamine system and mitochondrial function of dopaminergic neurons in a rotenone-induced rat model of PD. We found that chronic treatment with fucoidan significantly reversed the loss of nigral dopaminergic neurons and striatal dopaminergic fibers and the reduction of striatal dopamine levels in PD rats. Fucoidan also alleviated rotenone-induced behavioral deficits. Moreover, the mitochondrial respiratory function as detected by the mitochondrial oxygen consumption and the expression of peroxisome proliferator-activated receptor gamma coactivator 1-alpha (PGC-1α) and nuclear transcription factor 2 (NRF2) were reduced in the substantia nigra of PD rats, which were markedly reversed by fucoidan. Oxidative products induced by rotenone were significantly reduced by fucoidan. Taken together, these results demonstrate that fucoidan possesses the ability to protect the dopamine system in PD rats. The neuroprotective effect of fucoidan may be mediated via reserving mitochondrial function involving the PGC-1α/NRF2 pathway. This study provides new evidence that fucoidan can be explored in PD therapy.

Parkinson’s disease (PD) is the most common neurodegenerative movement disorder characterized pathologically by the progressive loss of dopaminergic neurons in the substantia nigra pars compacta (SNpc). This nigral neuronal loss consequently results in dopamine (DA) deficiency in the striatum, which is correlated with motor deficits such as tremor, rigidity and bradykinesia. The precise mechanisms underlying the pathogenesis and symptomology of PD are poorly understood. Available evidence supports that mitochondrial dysfunction plays a vital role in the development of PD [1].

The mitochondrion is a main energy-producing organelle in the cell. This organelle is actively involved in the regulation of a variety of cellular activities such as cell metabolism, intracellular calcium homeostasis, apoptosis, and free radical scavenging [2]. Due to the particular morphology and metabolism, dopaminergic neurons in the SNpc are considerably vulnerable to mitochondrial dysfunction [3-5]. The first evidence that linked mitochondrial dysfunction to PD was reported in an observation that exposure to 1-methyl-4-phenyl-1,2,3,6-tetrahydropyridine (MPTP), a mitochondrial complex I inhibitor, led to a parkinsonian syndrome in young drug abusers [6]. Subsequent studies reported that the parkinsonian features were reproduced in both primate and murine models treated with MPTP or other neurotoxins that inhibit the mitochondrial complex I [7, 8]. The post-mortem analysis of mitochondrial complex I deficiency in the SNpc of PD patients provided the direct relation between mitochondrial dysfunction and PD [9]. Recent report demonstrated that gene mutations associated with familial PD either directly or indirectly interfaced with pathways regulating mitochondrial function, thereby adding additional evidence for the role of mitochondrial dysfunction in PD [10-12]. Given the impaired mitochondrial function consistently in these studies, reserving the mitochondrial function may represent an effective strategy for the prevention and treatment of PD [13].

Fucoidan is a fucose-based sulfated polysaccharide extracted from brown algae (*Laminaria japonica*). It has a wide range of biological activities, including anti-inflammatory, antioxidant, antitumor, and immunomodulatory actions [14]. In previous studies, we have found that fucoidan exhibited the protective effect on dopaminergic neurons in MPTP-treated mice and lipopolysaccharide- and 6-hydroxydopamine-treated rats [15-17], establishing a potential of fucoidan in PD therapy. However, to date, detailed characteristics of, and molecular mechanisms underlying, this protective effect of fucoidan remain to be elucidated.

In the present study, we expanded the study of fucoidan to a rotenone-induced rat model of PD. We carried out a series of behavioral, morphological, and neurochemical experiments to characterize the time- and dose-dependent effect of fucoidan on pathological changes in rotenone-lesioned PD rats. Moreover, we investigated whether fucoidan exerts the neuroprotective effect on mitochondrial function of nigral dopaminergic neurons in response to DA lesions by rotenone.

## MATERIALS AND METHODS

### Materials and chemicals

Fucoidan was extracted from *Laminaria japonica* commercially cultured in Qingdao, China, as previously reported [18]. Its chemical composition was as follows: 48% total sugar content, 28% fucose content, and 29% sulfate content. Neutral monosaccharide analysis of fucoidan indicated that fucose was the main component with its molar ratio to galactose being 1:0.24. The average molecular weight of fucoidan was ~7 kDa determined by high performance steric exclusion chromatography analysis. Rasagiline was obtained from Tocris Bioscience (Atlantic Road, Bristol, UK). Rotenone and other reagents for measuring mitochondrial respiratory function were obtained from Sigma-Aldrich (St. Louis, MO, USA) except antimycin A from Biovision (St. Heinrich, Zurich, Switzerland) and oligomycin from Sellerk (Houston, TX, USA).

### Animals

Male Sprague-Dawley rats weighing 240-270 g were obtained from the laboratory animal center at Capital Medical University (Beijing, China). The animals were housed in an animal facility equipped with a standard 12 h on/off light cycle at 22°C and 60% humidity with food and water *ad libitum*. All animal experimental procedures were conducted according to the Ethics Committee on Animal Care and Use of Capital Medical University.

### Experimental design

Animals were randomly divided into seven groups and underwent a course of 38 days of treatments. Rats in group 1 received 0.9% saline for 10 days and were then co-treated with a vehicle solution, dimethyl sulfoxide (DMSO) + sunflower oil, for 4 weeks. This group of rats served as a vehicle control. Rats in group 2 received fucoidan (140 mg/kg/d) alone once a day for the entire 38 days. Rats in group 3 received rotenone (1.5 mg/kg/d) from day 11 (5 times a week for 4 weeks) and served as a model group. Rats in groups 4-6 received fucoidan at 35, 70, and 140 mg/kg/d respectively for 10 days and were then co-treated with rotenone for 4 weeks. Rats in group 7 received rasagiline for 10 days and were then co-treated with rotenone for 4 weeks, and served as a positive control. During a 4-week co-treatment period in groups 1 and 4-7, saline, different doses of fucoidan and rasagiline were administrated once a day, while rotenone and DMSO + sunflower oil were given 5 times a week.

**Figure 1:**
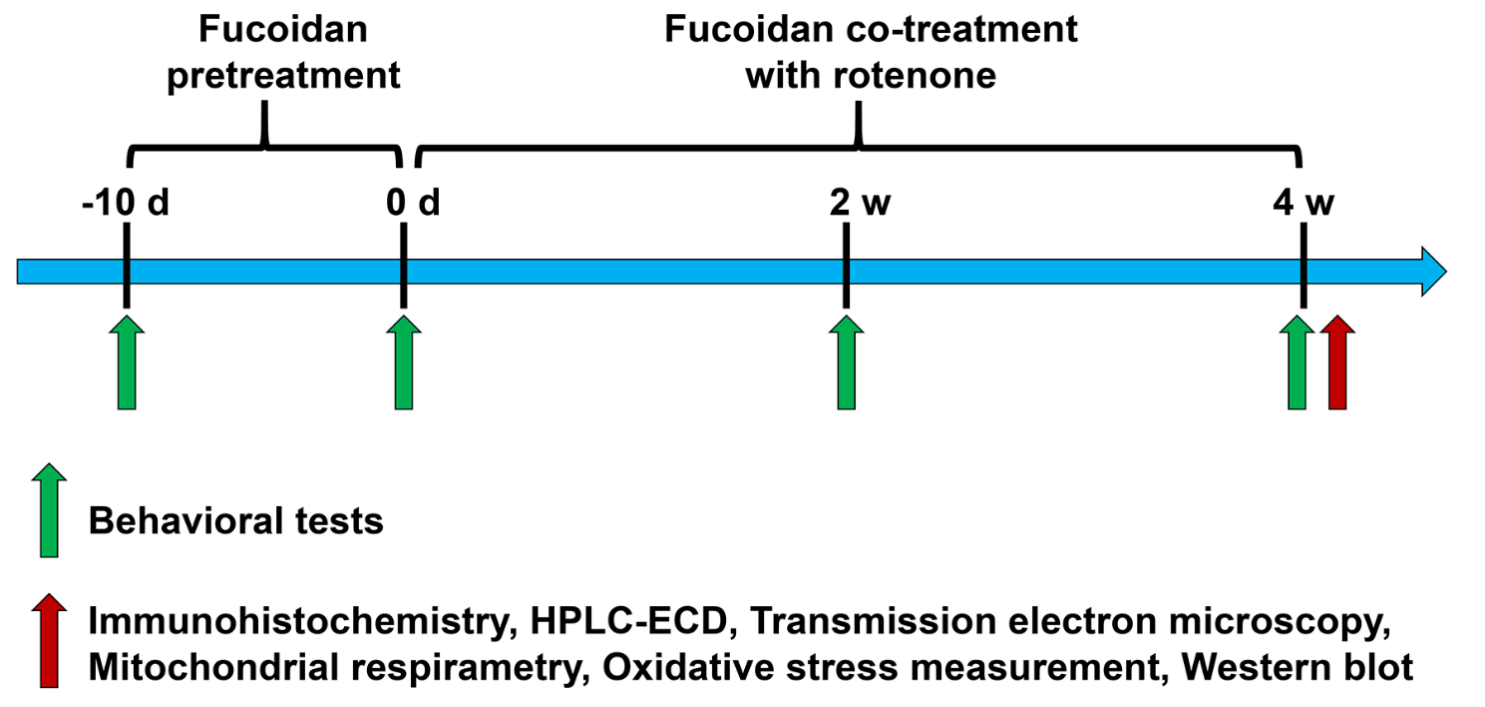
A schematic diagram of experiment arrangements Rats were pretreated with fucoidan for 10 days (once daily). Animals were then co-treated with fucoidan (once daily) and rotenone (5 times a week) for 4 weeks. Behavioral tests were conducted 1 day before fucoidan pretreatment, and 1 day before and 2 and 4 weeks after rotenone co-treatment with fucoidan or rasagiline. Other neurochemical assays, including immunohistochemistry, western blot, electron microscopy, HPLC-ECD analysis, mitochondrial respirometry and oxidative stress measurement were performed 1 day after a 4-week co-treatment period.

Fucoidan and rasagiline were dissolved in 0.9% saline and 0.5% hydroxypropyl methyl cellulose, respectively. Rotenone was dissolved in DMSO and diluted in sterile sunflower oil (2% final DMSO concentration and 98% final sunflower oil concentration). Fucoidan, rasagiline, and saline were given by gavage. Rotenone and the vehicle solution (DMSO + sunflower oil) were given by a subcutaneous injection. All drugs were administered at 2 ml/kg/d. Detailed experimental procedures were shown in Figure 1.

### Behavioral tests

Behavioral tests (from 8:00 a.m. to 6:00 p.m.) were performed 1 day before fucoidan pretreatment and 1 day before and 2 and 4 weeks after rotenone co-treatment with fucoidan or rasagiline.

#### Catalepsy

The cataleptic behavior was measured by the bar test and the grid test as previously described [19]. Briefly, in the bar test, rats were placed with the two front paws on a horizontal bar 9 cm above and parallel to the base in a half-rearing position. In the grid test, rats were hung by the paws on a vertical grid (25.5 cm wide and 44 cm high with a space of 1 cm between each wire). When the animals removed one paw from the bar or the grid, the time was recorded as descent latency (s). The maximum descent latency for catalepsy test was fixed at 180 s. Both tests were repeated 3 times. The mean values were used for further analysis.

#### Open-field test

The locomotor activity was investigated in automated activity chambers connected to a Digiscan analyzer that transmitted the number of beam breaks to a computer (Truscan 2.0 Instruments, Columbus, OH). The rats were initially placed in the center of the open field. Activity was then monitored for 30 min by a camera installed above the box. The locomotor activities were automatically calculated by a video tracking system using the same settings for all rats, and measured include floor plane (FP) movements, moving time (s), moving distance (cm), and mean velocity (cm/s).

### Immunohistochemistry and imaging quantification

Rats were anaesthetized with 10% chloral hydrate and perfused transcardially with 0.9% saline followed by 4% cold paraformaldehyde (PFA). Brains were removed, postfixed overnight in 4% PFA at 4?, and then placed in 20% and 30% sucrose in phosphate buffered saline (PBS) for cryoprotection. Coronal sections containing the striatum (30 μm) and the substantia nigra (SN, 50 μm) were cut by a Leica freezing microtome (Solms, Germany). A sequence of incubation steps was done in 0.3% Triton-X for 30 minutes, then 3% hydrogen peroxide for 30 minutes, and rinsed with PBS (3 times for 10 minutes each) between each step. After blocked with 5% normal goat serum (Vector Laboratories) in 0.1 M PBS for 1 h at room temperature, sections were incubated overnight with a mouse antibody against tyrosine hydroxylase (TH, 1:2000, AMAB91112). The antibody was detected using an ABC Elite kit (Vector laboratories, Burlingame, CA, USA) with a 3,3′-diaminobenzidine solution.

The number of TH-positive neurons in the bilateral SNpc was counted stereologically using an optical fractionator method in a computerized system (Stereo Investigator, Leica Microsystems CMS GmbH). Every sixth section through the entire SN was sampled. A total of 8 sections per animal were collected for the analysis. The estimates of the total number of TH-positive neurons were calculated according to the optical fractionator formula and the Gundersen coefficients of error (m = 1) values were < 0.1 for all animals. The staining intensity of TH-immunoreactivity in the striatum was analyzed by densitometry using Image-J software to quantify striatal TH-positive terminal density. All sections were coded and examined blindly.

### Measurements of striatal DA and its metabolites

Levels of DA and its metabolites, 3,4-dihydroxyphenylacetic acid (DOPAC) and homovanillic acid (HVA), in the striatum were analyzed using high performance liquid chromatography with an electrochemical detector (HPLC-ECD) (Model 5600A Coul Array Detector System ESA, Brighton, MA, USA) according to a previously described method [16]. Briefly, frozen striatal samples were homogenized in 0.2 M ice-cold perchloric acid. The homogenates were placed in an ice bath for 60 min. Subsequently, the samples were centrifuged at 15000 g for 20 min at 4°C and the supernatant was transferred to a clean tube. One-half volume of a solution containing 0.02 M potassium citrate, 0.3 M KH_2_PO_4_, and 0.002 M Na_2_EDTA was added and mixed thoroughly to deposit perchloric acid. After incubation for 60 min, the mix was centrifuged at 15000 g for 20 min at 4°C. The supernatant was collected, filtered through a 0.22 µm millipore filter, and injected into the HPLC system. The mobile phase was 125 mM sodium citrate buffer containing 20% methanol, 0.1 mM EDTA·2Na, and 0.5 mM 1-octanesulfonic acid sodium salt (Acros Organics, NJ, USA) and adjusted to pH 4.3. The flow rate was set at 1.2 ml/min. Values were normalized to the wet weight of the samples.

### Transmission electron microscopy

Rats were anesthetized with chloral hydrate and perfused transcardially with 2% glutaraldehyde and 2% PFA in 0.1 M PBS, pH 7.4. Brains were removed and postfixed in the same fixative for 12 h. They were then vibratome sectioned coronally (200 μm) at the level of the midbrain. Small tissue blocks (1 mm^2^) excised from the SNpc area on the sections were postfixed, dehydrated, and resin-embedded as descried previously [20]. The samples were imaged with a transmission electron microscope (Hitachi, Japan). Imaging analysis using 10000× magnification was carried out by an investigator blinded to the experimental groups.

### In situ studies of mitochondrial respiratory function

The mitochondrial respiratory function was assayed by measuring oxygen consumption rates (OCRs). We used a real time high-resolution respirometry (Oxygraph-2k; Oroboros Instruments, Innsbruck, Austria) under a variety of substrate conditions and respiratory states to examine differences in respiratory capacity as described previously [21]. The oxygen concentration was measured by a Clark electrode. DatLab software was employed for data acquisition and analysis. OCRs were measured, normalized to the protein concentration, and expressed as pmol of O_2_ per second per mg protein.

The ventral midbrain of rats was ground in a grinding pestle and suspended in 2.1 ml mitochondrial respiration medium MiR05 containing 0.5 mM EGTA, 3 mM MgCl_2_.6H_2_O, 60 mM K-lactobionate, 20 mM taurine, 110 mM sucrose, 1 g/L bovine serum albumin (essentially fatty acid-free), 10 mM KH_2_PO_4_, and 20 mM 4-(2-hydroxyethyl)-1-Piperazineethanesulfonic acid. KOH (5 N) was used to adjust pH to 7.1. The assay was maintained at 37? and 750 rpm stirrer speed. A number of parameters were measured by a sequence of titration, including 4 mg/ml oligomycin (2 µl), 1 mM tri-fluorocarbonylcyanide phenylhydrazone (FCCP) (1 µl), and 1 mM rotenone + 5 mM antimycin A (1 µl). In addition to determinations of basal respiration, adenosine triphosphate (ATP) production was calculated by basal respiration minus respiratory oxygen consumption under 4 mg/ml oligomycin. Maximal respiration was determined by uncoupling respiration with 1 µM FCCP. Residual oxygen consumption was measured by adding 1 mM rotenone and 5 mM antimycin A.

A panel of substrates and inhibitors, including pyruvate + malate, adenosine diphosphate (ADP), glutamate, rotenone, succinate and antimycin A, was titrated sequentially in digitonin-permeabilized tissue homogenates to assay the function of mitochondria complex I and II. The peak function of complex I is measured by adding 0.8 M malate + 2 M pyruvate (2 µl), 0.5 M ADP (4 µl), and 2 M glutamate (2 µl) (substrates of complex I). Complex I-associated respiration (CI) is measured as the drop, from this peak, induced by adding 0.1 mM rotenone (1 µl). Subsequently, complex II-associated respiration (CII) was measured by determining the difference between 1 M succinate (1 µl)-induced respiration and the residual respiration after adding 5 mM antimycin A (1 µl). Titration of 10 µM cytochrome c (1 µl) was used for the assessment of mitochondrial membrane integrity. Preliminary experiments were performed to determine the concentration of these agents which could optimally inhibit or stimulate respiration without inducing toxicity.

**Figure 2:**
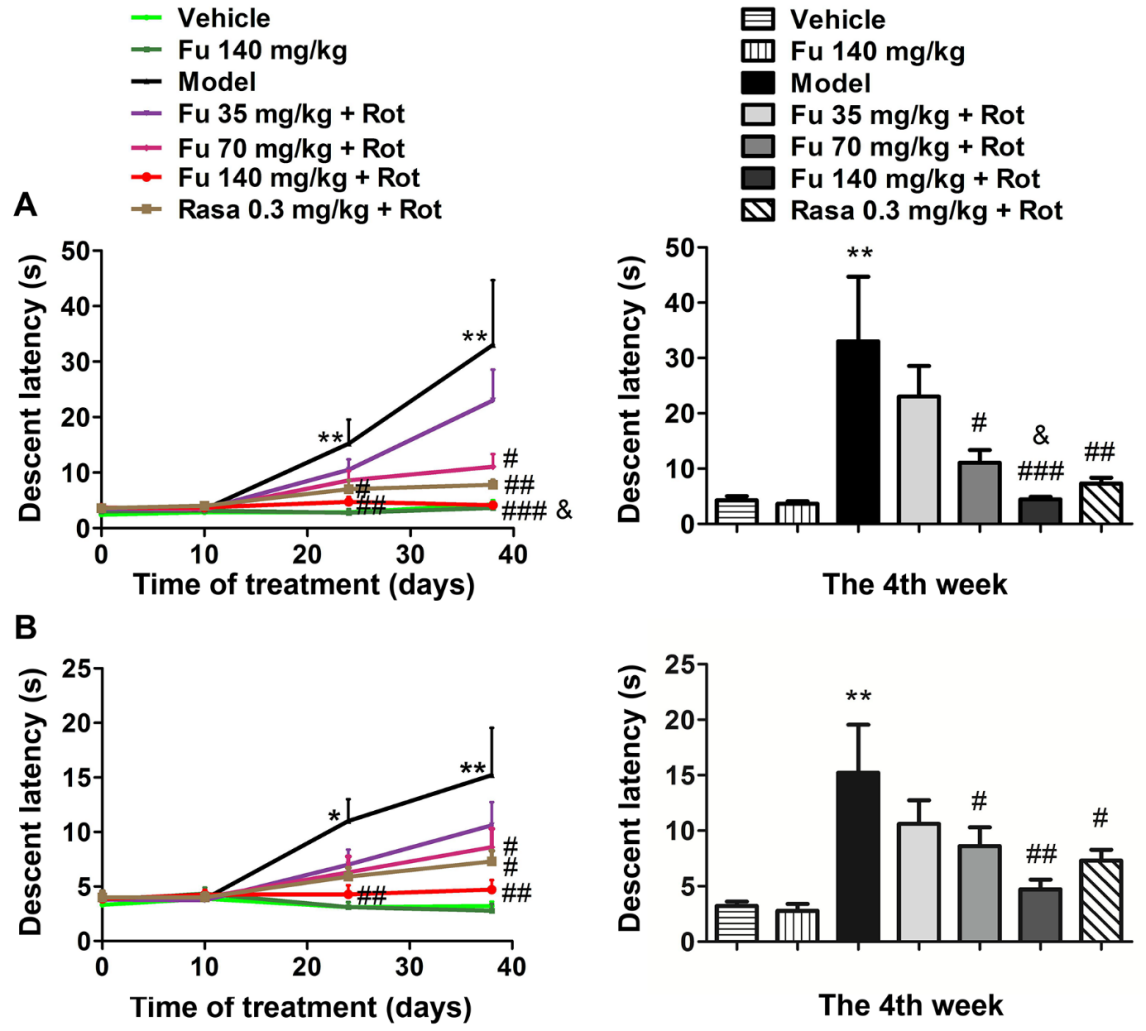
Effects of fucoidan on rotenone-induced catalepsy in rats (**A** and **B**) Effects of fucoidan on rotenone-induced catalepsy as detected by a bar test (**A**) and a grid test (**B**). Data collected at the fourth week were quantified in the right panels. Note that fucoidan (Fu) dose-dependently reduced cataleptic responses to rotenone (Rot). Comparison between the 140 mg/kg/d fucoidan group and the 0.3 mg/kg rasagiline (Rasa) group yields *P* < 0.05 for the bar test. Data are shown as means ± SEM (n = 9-12 per group). *P < 0.05 and **P < 0.01 versus vehicle group at the same time point. ^#^*P* < 0.05, ^##^*P* < 0.01, and ^###^*P* < 0.001 versus model group (rotenone only) at the same time point. ^&^*P* < 0.05 versus rasagiline group at the fourth week.

**Figure 3:**
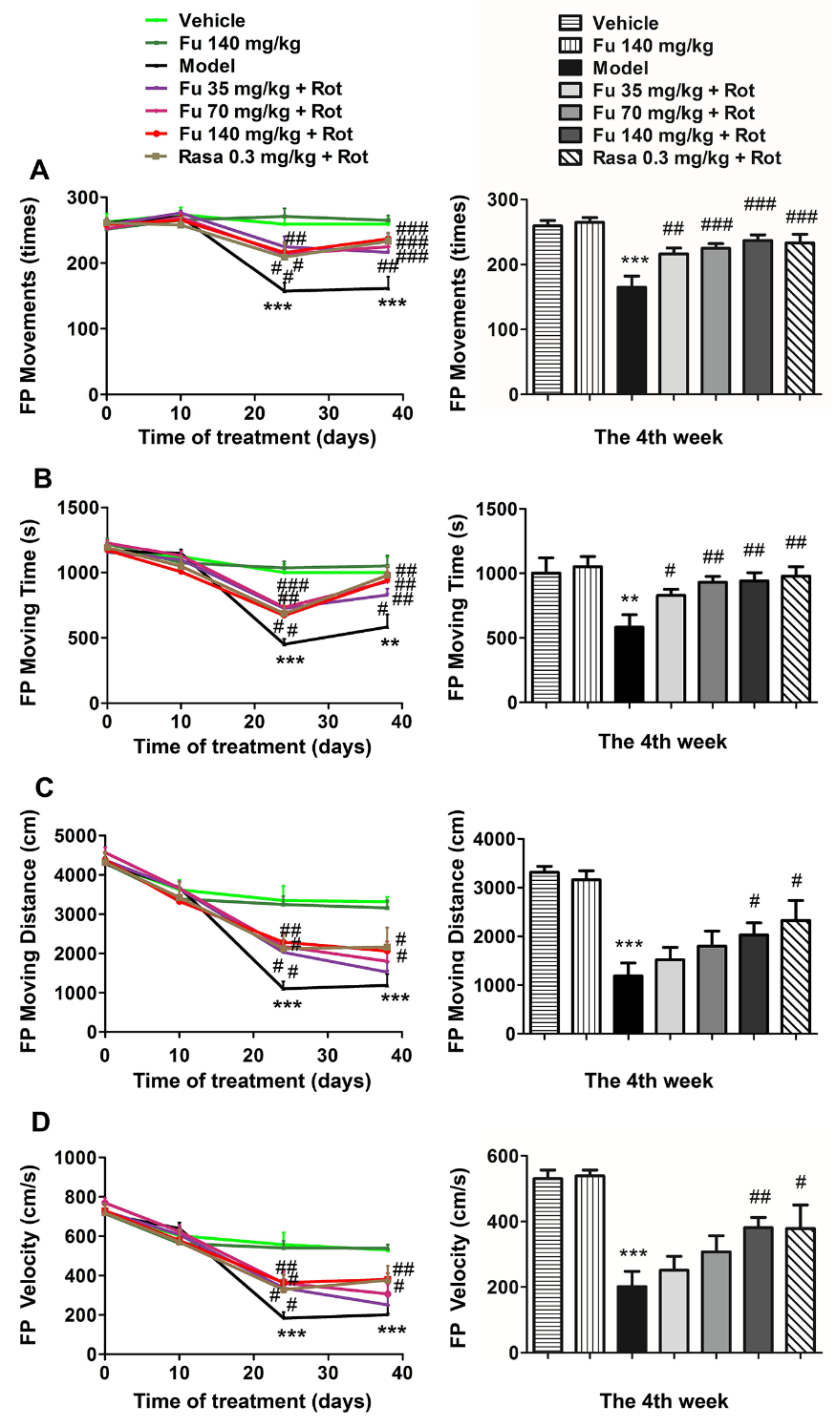
Effects of fucoidan on rotenone-induced reduction of locomotor activity in rats (**A-D**) Effects of fucoidan on the rotenone-induced reduction in floor plane (FP) movements (**A**), moving time (**B**), moving distance (**C**), and mean velocity (**D**). Note that fucoidan reversed the reduction of all four types of locomotor activities. Data are shown as means ± SEM (n = 9-12 per group). **P < 0.01 and ***P < 0.001 versus vehicle group at the same time point. ^#^*P* < 0.05, ^##^*P* < 0.01, and ^###^*P* < 0.001 versus model group (rotenone only) at the same time point.

### Measurements of oxidative stress products

A commercial kit (Jiancheng Institute of Biotechnology, Nanjing, China) was used to measure the concentration of malondialdehyde (MDA) in the ventral midbrain according to the manufacturer’s instructions. Similarly, levels of 3-nitrotyrosine (3-NT) and 8-hydroxy-2-deoxyguanosine (8-OHdG) were determined using an enzyme-linked immunosorbent assay kit (CUSABIO BIOTECH CO., Ltd., Wuhan, China) by following the manufacturer’s instructions.

### Western blot analysis

Tissue was lysed in RIPA buffer (Beyotime, Beijing, China) containing a protease inhibitor cocktail (Roche, Mannheim, Germany). The protein concentration was measured by a BCA method. Proteins were run on sodium dodecyl sulfate polyacrylamide gel electrophoresis and then transferred to polyvinylidene fluoride membranes (Millipore, MA, USA). After blocking, membranes were incubated overnight at 4? with an antibody against peroxisome proliferator-activated receptor gamma coactivator 1-alpha (PGC-1α; 1:1000, ab191838, Abcam, Cambridge, MA, USA), nuclear respiratory factor (NRF2; 1:1000, #12721, Cell Signaling Technology, Danvers, MA, USA), or β-actin (1:5000, A1978, Sigma-Aldrich). After washing, membranes were incubated with a secondary antibody (1:10000, Rockland Immunochemicals, Gilbertsville, PA, USA) for 1 h and scanned with an Odyssey infrared imaging system (LI-COR instrument, Lincoln, NE, USA). Immunoblots were quantified using Image J software and values were normalized to β-actin.

**Figure 4:**
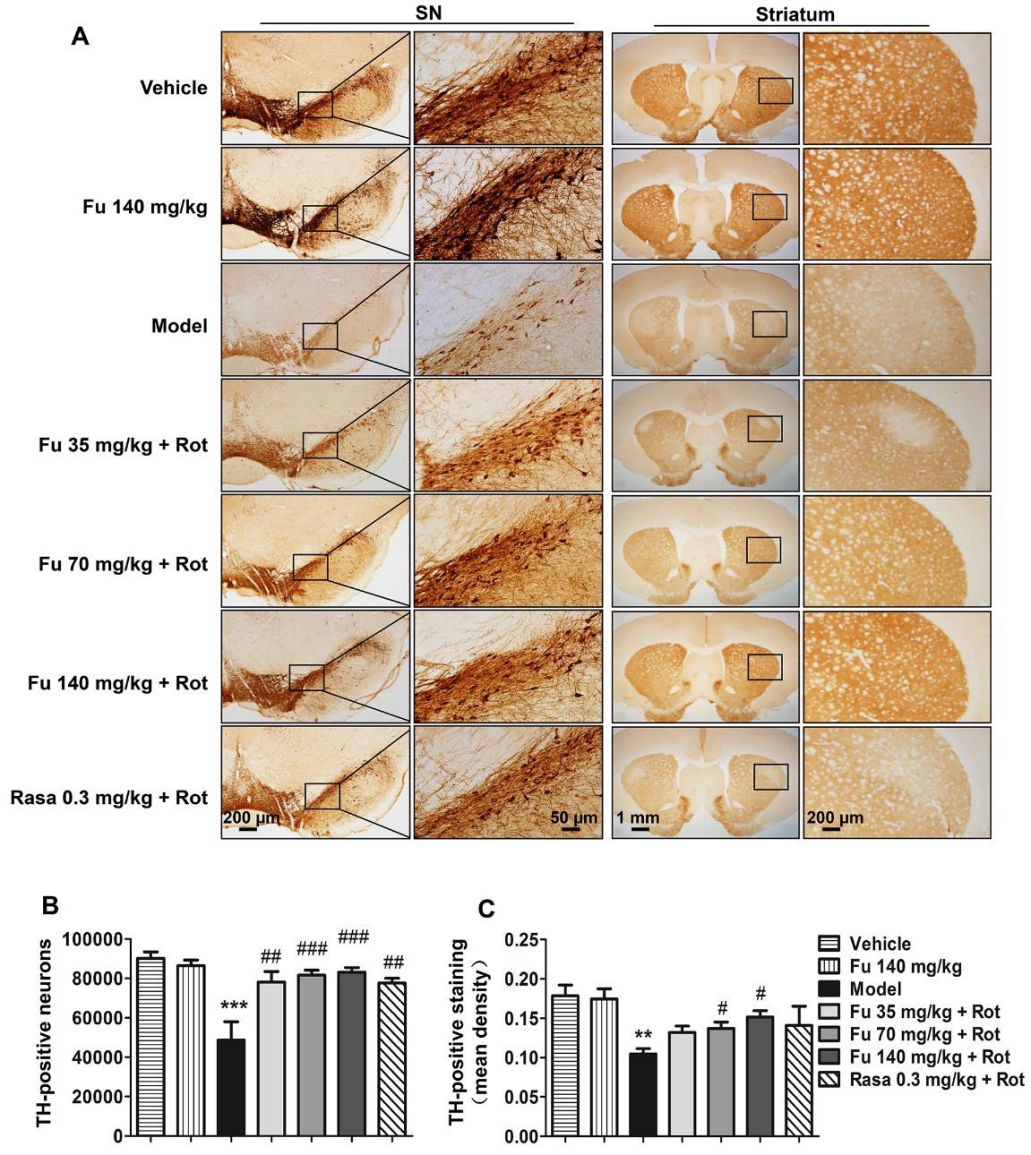
Effects of fucoidan on the rotenone-induced loss of TH-positive neurons and fibers in the nigrostriatal system (**A**) Representative immunohistochemical images depicting changes in TH immunoreactive neurons and fibers in the SNpc and striatum, respectively. (B and C) Quantifications of the number of nigral TH-positive neurons (**B**) and the mean density of striatal TH-positive fibers (**C**). Note that rotenone caused a loss of TH-positive neurons in the SNpc and TH-positive fibers in the striatum, and fucoidan was able to reverse these losses. Data are shown as means ± SEM (n = 3-5 per group). **P < 0.01 and ***P < 0.001 versus vehicle group. ^#^*P* < 0.05, ^##^*P* < 0.01, and ^###^*P* < 0.001 versus model group (rotenone only).

### Statistical analysis

All statistical analyses were processed using Prism 6.0 software. Data are expressed as means ± SEM. One-way analysis of variance (ANOVA) analysis followed by Newman Keuls *post-hoc* analysis was used for comparison among multiple groups. Student’s *t*-test was used to compare data between two groups. *P* < 0.05 was considered as statistically significant.

**Figure 5:**
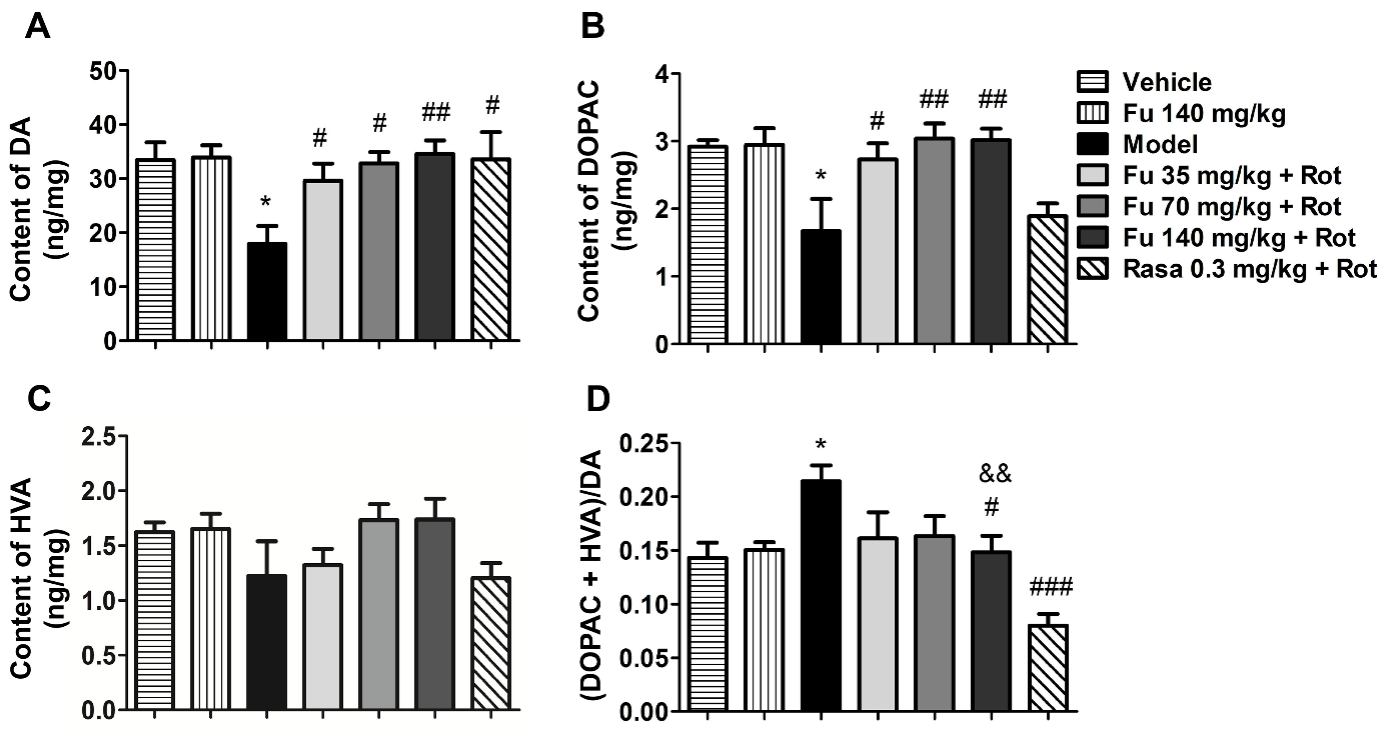
Effects of fucoidan on contents of striatal DA and its metabolites (**A-C**) Effects of fucoidan on striatal DA, DOPAC, and HVA levels. (**D**) Effects of fucoidan on the ratio of DOPAC + HVA to DA. The contents of DA, DOPAC, and HVA in the striatum were measured by HPLC. Note that fucoidan reversed a decrease in DA and DOPAC levels and an increase in the ratio of DOPAC + HVA to DA induced by rotenone. The difference of DA turnover rate between the 140 mg/kg fucoidan group and 0.3 mg/kg rasagiline group was significant. Data are shown as means ± SEM (n = 3-5 per group). *P < 0.05 versus vehicle group. ^#^*P* < 0.05, ^##^*P* < 0.01, and ^###^*P* < 0.001 versus model group (rotenone only). ^&&^ *P* < 0.01 versus rasagiline group.

## RESULTS

### Effects of fucoidan on catalepsy in PD rats

As measured by the descent latency in the bar (Fig. 2A) and grid test (Fig. 2B) during the acquisition trials at four different time points, rats in the model group displayed a progressive increase in the descent latency as compared to the control group (vehicle only). Remarkably, pretreatment with fucoidan significantly prevented the increase in the descent latency. The effect of fucoidan was in a dose-dependent manner (35, 70, and 140 mg/kg). At the fourth week, while a lower dose of fucoidan (35 mg/kg) was ineffective, fucoidan at the two higher doses (70 and 140 mg/kg) significantly reduced the descent latency. As expected, the monoamine oxidase inhibitor rasagiline also ameliorated the cataleptic behavior induced by rotenone (seventh group), which served as a positive control. Notably, fucoidan at 140 mg/kg reduced the descent latency in bar test to a level lower than that seen in the rasagiline group. Fucoidan alone did not result in cataleptic behavior in normal rats.

### Effects of fucoidan on locomotor activity in PD rats

A variety of locomotor activities was measured in this study, which include FP movements, moving time, moving distance, and mean velocity. As shown in Figure 3, all four types of locomotor activities consistently underwent a marked decrease in rotenone-treated rats relative to vehicle-treated rats. Pretreatment with fucoidan and rasagiline significantly alleviated the decrease in four locomotor activities. In details, fucoidan at three doses (35, 70, and 140 mg/kg) significantly reversed a decrease in FP movements (Fig. 3A) and FP moving time (Fig. 3B). Fucoidan at a higher dose (140 mg/kg) although not at the two lower doses (35 and 70 mg/kg) reversed a decrease in FP moving distance (Fig. 3C) and FP mean velocity (Fig. 3D). The effects of 70 and 140 mg/kg fucoidan on FP movements and moving time, and 140 mg/kg fucoidan on moving distance and mean velocity were similar to that of rasagiline after four-week treatment. Fucoidan alone did not affect the locomotor activity of normal rats.

**Figure 6:**
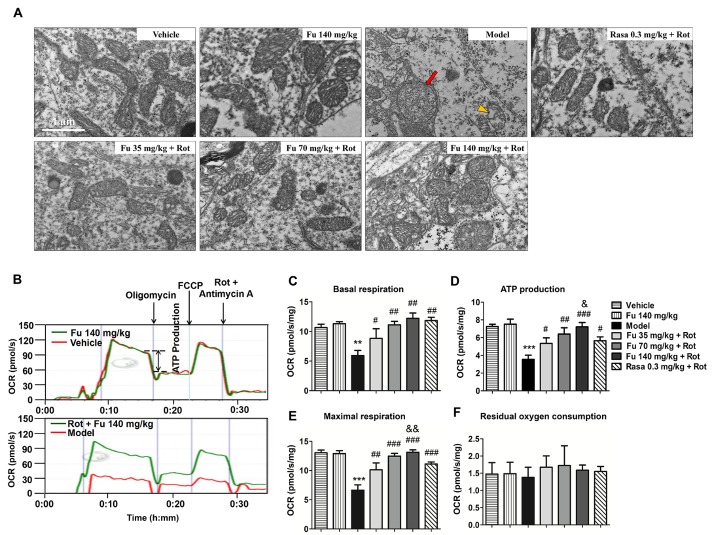
Effects of fucoidan on rotenone-induced alterations of mitochondrial morphology and respiration function in the rat ventral midbrain (**A**) Electron microscopic images illustrating morphological changes in the mitochondria in the rat SNpc. (**B**) Representative recordings of mitochondrial respiration. (**C-F**) Quantification of basal respiration (**C**), ATP production (**D**), maximal respiration (**E**), and residual oxygen consumption (**F**) in the ventral midbrain of rats. Data are shown as means ± SEM (n = 3-4 per group). **P < 0.01 and ***P < 0.001 versus vehicle group. ^#^*P* < 0.05, ^##^*P* < 0.01, and^ ###^*P* < 0.001 versus model group (rotenone only). ^&^*P* < 0.05 and^ &&^*P* < 0.01 versus rasagiline group. Scale bar = 1μm.

### Effects of fucoidan on the loss of nigral TH-positive neurons and striatal TH-positive fibers in PD rats

To determine whether fucoidan protects against the rotenone-induced loss of nigral DA neurons and striatal DA fibers, we performed immunohistochemistry to monitor changes in TH immunoreactivity in the SNpc and striatum. Rotenone produced a marked loss of TH-positive neurons in the SNpc. Rotenone also induced a significant reduction of TH-positive fibers in the striatum. Pretreatment with fucoidan at the three doses reduced the loss of TH-positive neurons in the SNpc. Fucoidan at least at the two higher doses (70 and 140 mg/kg) also reduced the loss of TH-positive fibers in the striatum. The effect of 35 mg/kg fucoidan on the loss TH-positive neurons and fibers was similar to that of rasagiline. Fucoidan alone (the second group) had a minimal impact on TH-positive neurons in the SNpc and TH-positive fibers in the striatum (Fig. 4A). Quantification of TH-positive neurons and fibers in the respective SNpc (Fig. 4B) and striatum (Fig. 4C) confirmed the image impression.

### Effects of fucoidan on contents of striatal DA and its metabolites in PD rats

Changes in the amount of DA and its metabolites in the striatum were measured by HPLC-ECD. Rotenone alone resulted in a significant decrease in DA (Fig. 5A) and DOPAC (Fig. 5B). While the concentration of HVA remained unchanged in rotenone-treated rats compared to vehicle-treated rats (Fig. 5C), the turnover rate of DA which was calculated by the ratio of (DOPAC + HVA) to DA was significantly increased (Fig. 5D). Notably, fucoidan at the three doses prevented the reduction of DA and DOPAC induced by rotenone. Moreover, fucoidan at 140 mg/kg reversed the increase in the turnover rate of DA in response to rotenone. Although the effect was lower than that of 0.3 mg/kg rasagiline, DA turnover rate of rats pretreated with fucoidan was similar to that of normal rats. Fucoidan alone did not alter the levels of striatal DA and its metabolites.

**Figure 7:**
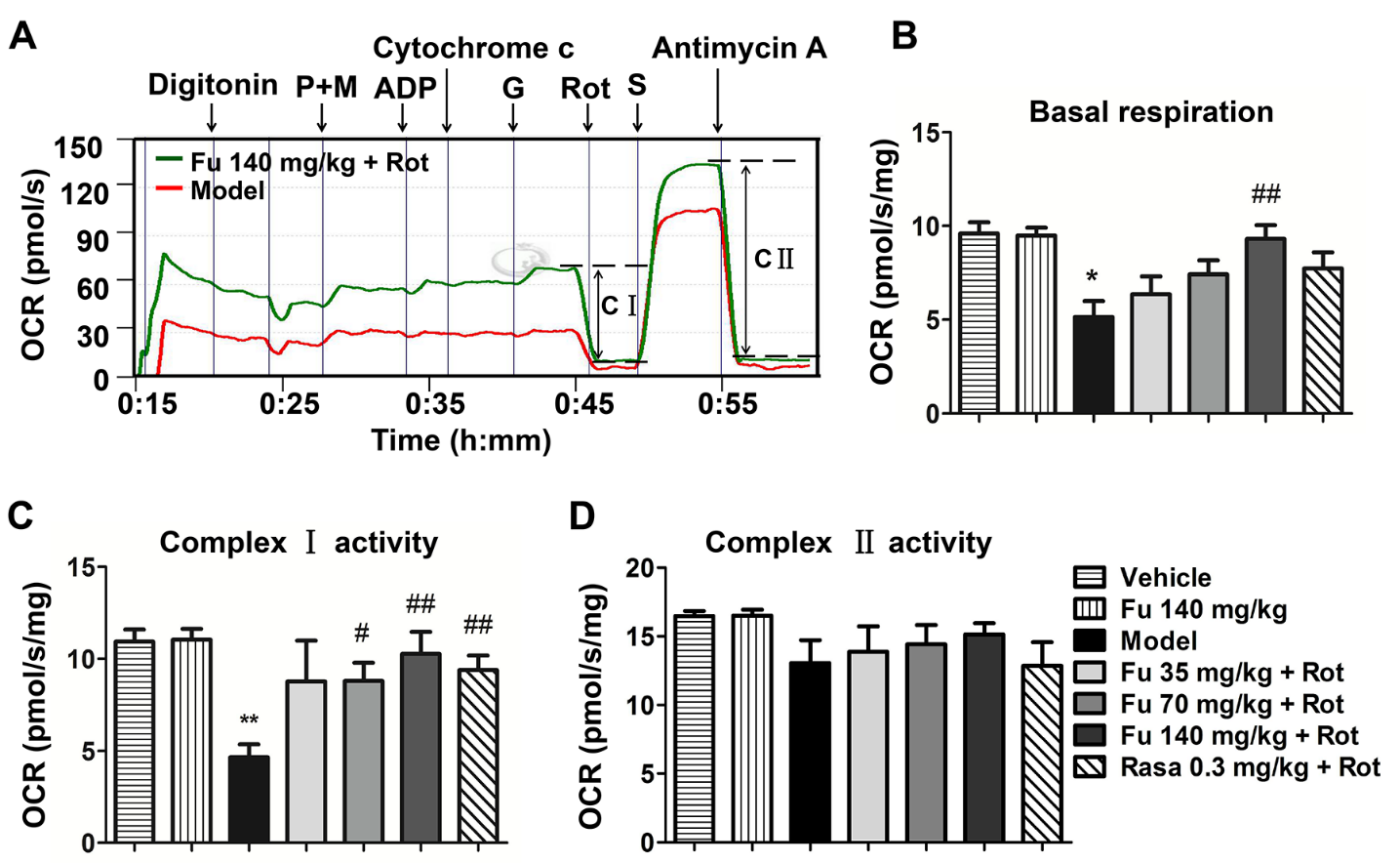
Effects of fucoidan on rotenone-induced reduction of mitochondrial complex activity in the rat ventral midbrain (**A**) Profiles of OCRs in the digitonin-permeabilized ventral midbrain tissue subjected to rotenone (red line) or fucoidan with rotenone (green line). (B-D) Quantification of basal respiration (**B**), complex I activity (**C**), and complex II activity (**D**) in the ventral midbrain of rats. Data are shown as means ± SEM (n = 3-4 per group). *P < 0.05 and **P < 0.01 versus vehicle group. #*P* < 0.05 and ##*P* < 0.01 versus model group (rotenone only). Abbreviation: P + M = pyruvate + malate, G = glutamate, S = succinate.

### Effects of fucoidan on mitochondrial morphology and respiratory capacity in PD rats

The ultrastructure of the mitochondria in the soma of neurons located in the ventral midbrain was observed by electron microscopy. As shown in Figure 6A, the mitochondria exhibited normal structures with a dense matrix and regular cristae in rats treated with vehicle or fucoidan alone. In contrast, the mitochondria of rotenone-treated rats were swollen with reduced fragmented cristae (red arrow) and a little to no matrix (yellow arrowhead). In rats co-treated with fucoidan and rotenone, the mitochondria were relatively normal and were featured with rod-like, dense, and organized cristae.

**Figure 8:**
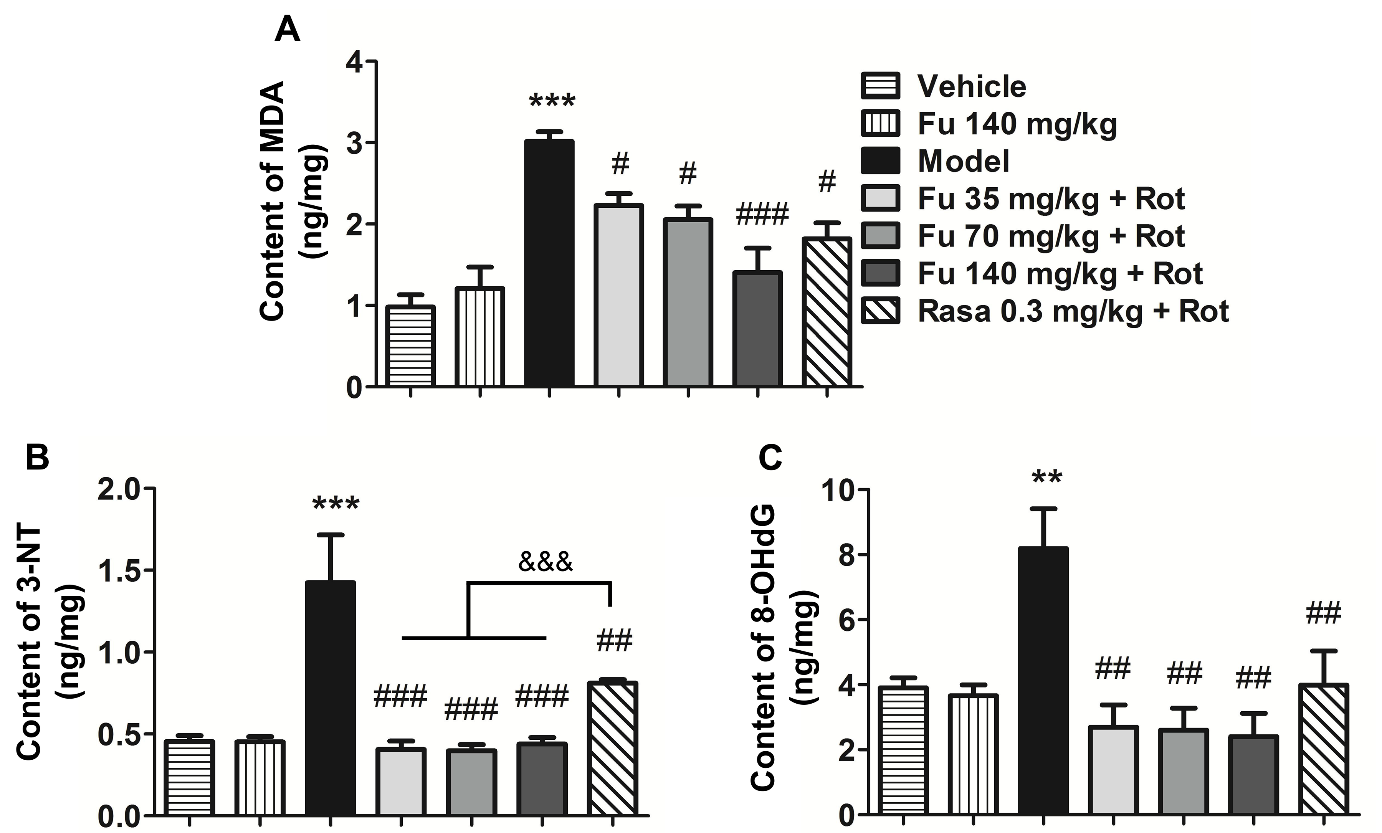
Effects of fucoidan on oxidative stress responses to rotenone in the rat ventral midbrain (**A-C**) Effects of fucoidan on the rotenone-induced increases in MDA (**A**), 3-NT (**B**), and 8-OHdG (**C**) levels in the ventral midbrain of rats. Data are shown as means ± SEM (n = 4-5 per group). **P < 0.01 and ***P < 0.001 versus vehicle group.^ #^*P* < 0.05,^ ##^*P* < 0.01 and ^###^*P* < 0.001 versus model group (rotenone only). ^&&&^ *P* < 0.001 versus rasagiline group.

The mitochondrial respiratory function was assessed by analyzing a number of parameters including mitochondrial basal respiration, ATP production, maximal respiration and residual oxygen consumption. As illustrated in Figure 6B, fucoidan alone had no significant effect on mitochondrial respiratory function, while rats treated with rotenone alone exhibited the markedly reduced mitochondrial respiratory function. Fucoidan at the three doses mitigated the rotenone-induced decrease of basal respiration (Fig. 6C), ATP production (Fig. 6D) and maximal respiration (Fig. 6E) in a dose-dependent manner. Fucoidan at 140 mg/kg completely restored the levels of these three parameters, and the beneficial effect on ATP production and maximal respiration were even better than that of rasagiline. There were no significant differences in residual oxygen consumption between different groups (Fig. 6F), indicating that the effect of fucoidan on the respiratory function less likely results from nonmitochondrial oxygen consumption.

### Effects of fucoidan on mitochondrial complex activity in PD rats

In order to dissect the function of mitochondrial complex, we tested the activity of mitochondrial complex I and II in the permeabilized rat ventral midbrain tissue. As shown in Figure 7A-C, the basal respiration and complex I activity in the ventral midbrain tissue were reduced in rotenone-treated rats compared to vehicle-treated rats. Fucoidan at 140 mg/kg dramatically increased the basal respiration. Fucoidan at 70 and 140 mg/kg also increased complex I activity. The effect of 70 mg/kg fucoidan on basal respiration and complex I activity was similar to that of rasagiline. The mitochondrial complex II activity showed a tendency of decline in rotenone-treated rats, although it did not reach a statistically significant level (Fig. 7D). Fucoidan alone did not alter the basal respiration and the complex I and II activities in normal rats.

### Effects of fucoidan on levels of oxidative stress products in PD rats

The oxidative stress status was evaluated by measuring contents of three oxidative stress products, including MDA, 3-NT and 8-OHdG, in the rat ventral midbrain. Rotenone significantly increased levels of MDA (Fig. 8A), 3-NT (Fig. 8B), and 8-OHdG (Fig. 8C). In the presence of fucoidan, rotenone produced a significantly less increase in MDA levels. Fucoidan completely abolished the increases in 3-NT and 8-OHdG levels induced by rotenone, and the effect on 3-NT was even better than that of rasagiline. Fucoidan alone had no significant effect on basal levels of the three products in normal rats.

### Effects of fucoidan on PGC-1α and NRF2 protein expression in PD rats

**Figure 9:**
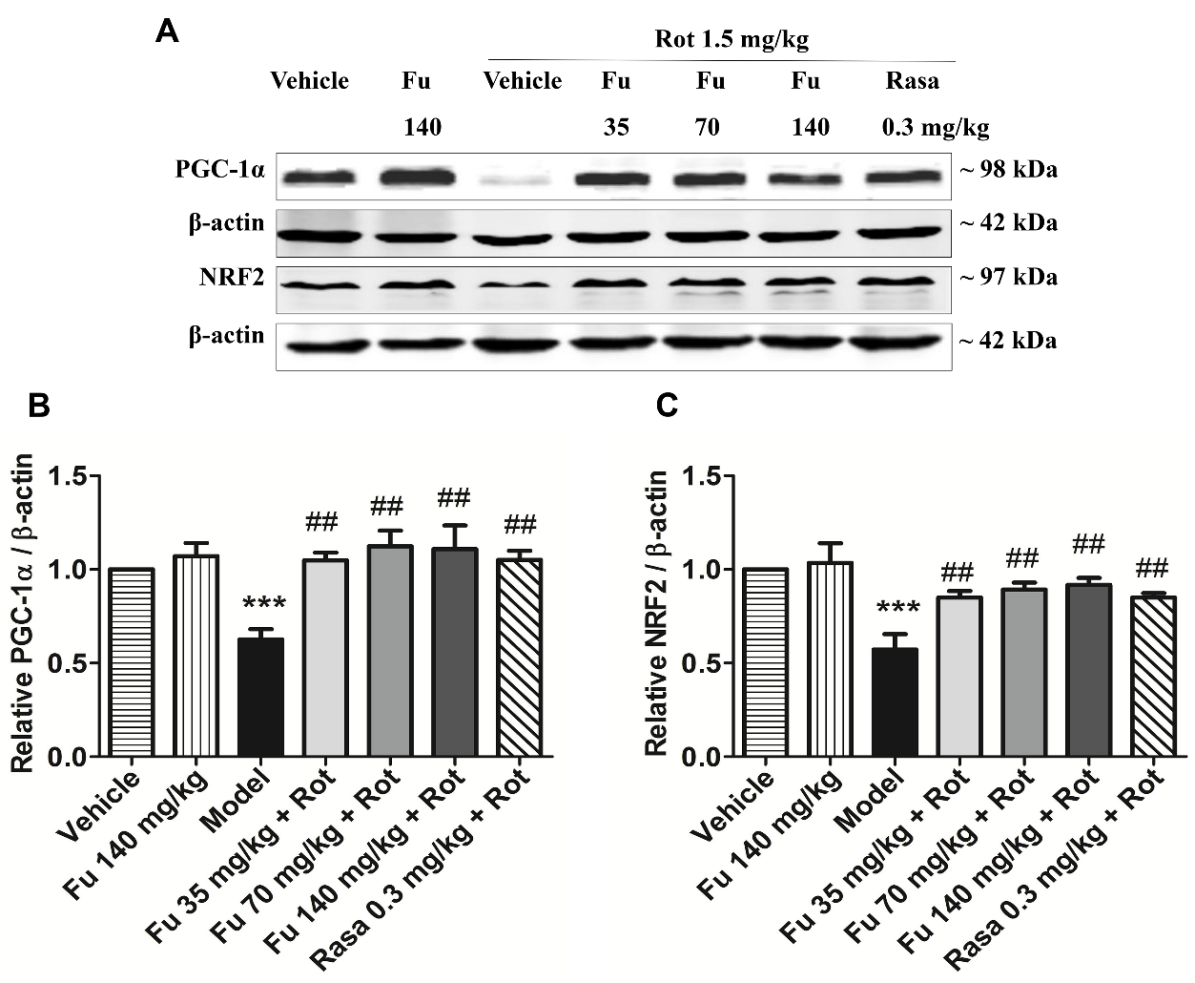
Effects of fucoidan on PGC-1α and NRF2 expression in the rat ventral midbrain (**A**) Representative immunoblots illustrating effects of fucoidan on PGC-1α and NRF2 expression in the ventral midbrain of rotenone-treated rats. (**B** and **C**) Quantification of PGC-1α (**B**) and NRF2 (**C**) expression in the ventral midbrain of rotenone-treated rats. Note that rotenone decreased PGC-1α and NRF2 expression in the ventral midbrain, which was reversed by fucoidan. Data are shown as means ± SEM (n = 3-4 per group). ***P < 0.001 versus vehicle group. ^##^*P* < 0.01 versus model group (rotenone only).

Western blot analysis was performed to determine changes in expression of PGC-1α and NRF2, two proteins related to mitochondrial function. As shown in Figure 9A and 9B, expression levels of PGC-1α in the ventral midbrain were downregulated in PD model rats (rotenone alone) relative to vehicle-treated rats. Fucoidan pretreated at the three doses significantly restored the level of PGC-1α. Similar results were observed in analyzing NRF2 expression (Fig. 9A and 9C). Rasagiline also restored the reduction of PGC-1α and NRF2 expression induced by rotenone. Fucoidan alone did not alter basal levels of PGC-1α and NRF2 proteins in the ventral midbrain of normal rats.

## DISCUSSION

As mitochondrial dysfunction has been considered to play an important role in dopaminergic neuron degeneration in both sporadic and familial PD patients, approaches directed to maintain mitochondrial function hold a great potential for prevention and treatment of PD. In the present study, fucoidan, a traditional Chinese medicine, was found to be potent to modify mitochondrial function. The results obtained from a complete set of experiments demonstrate that fucoidan profoundly rescued the mitochondrial respiratory function in rotenone-lesioned PD rats, while fucoidan at the same time substantially alleviated motor impairments and nigrostriatal dopaminergic degeneration in these rats.

Rotenone, a potent and specific mitochondrial complex I inhibitor, is a lipophilic pesticide widely used in agriculture. As it readily passes the blood brain barrier, systemic exposure to rotenone replicated the neurochemical, behavioral, and neuropathological features of PD [8]. In the present study, a dose of 1.5 mg/kg/d of rotenone for 4 weeks significantly impaired behavior as manifested by a progressive increase in catalepsy and a decrease in locomotor activity, which are thought to mimic the rigidity and bradykinesia in PD patients. These PD-like symptoms are reported to be closely associated with DA levels in the striatum, which is reduced in PD patients as a result of a loss of dopaminergic neurons in the SNpc. In this study, application of rotenone significantly decreased nigral DA neurons, striatal DA fibers and striatal DA level. Remarkably, fucoidan was able to reverse these decreases in the DA system. Thus, fucoidan can protect the dopaminergic pathway and improve behavioral deficits in PD animals. The DA level in the striatum is also determined by the DA catabolic rate which can be measured by the ratio of (DOPAC + HVA)/DA. In this study, an abnormal increase in the ratio of (DOPAC + HVA)/DA was seen in the striatum of PD rats, which was significantly inhibited by rasagiline. Fucoidan at 140 mg/kg also decreased the ratio, but the effect was significantly weaker than that of rasagiline. This suggests that different mechanisms may underlie the effect of two drugs. Rasagiline is a known monoamine oxidase B inhibitor which can increase the contents of DA in the striatum of PD patients through a mechanism involving the inhibition of DA catabolism [22].

Given the crucial role of mitochondrial dysfunction in the PD pathogenesis, we sought to investigate whether the neuroprotective effect of fucoidan was mediated through alleviating mitochondrial dysfunction. To probe mitochondrial dysfunction, we performed the real-time high-resolution respirometry with the ventral midbrain tissue, an assay which enables a standardized and specific analysis of mitochondrial respiratory function [23, 24]. Mitochondrial respiratory capacity is a critical determinant of cell survival especially under stressful conditions. Base respiration is used to characterize the aerobic activity of cell under ordinary condition. ATP production is used to describe the amount of ATP that can be produced by oxidative phosphorylation, while maximal respiration indicates the maximum ability of oxidative phosphorylation in case of a sudden increase in energy demand. We found that fucoidan prevented a decrease in mitochondrial respiratory function induced by rotenone. In this action, fucoidan at a dose of 140 mg/kg almost completely restored the levels of mitochondrial respiration, and the effect on ATP production and maximal respiration was even better than rasagiline. These results indicate that fucoidan can protect mitochondrial respiratory function against rotenone toxicity.

Mitochondrial complex I (nicotinamide adenine dinucleotide: ubiquinone oxidoreductase) is the upstream complex in the mitochondrial electron transport chain. It catalyzes the transfer of electrons from nicotinamide adenine dinucleotide to downstream molecules [25]. Several studies indicate that mitochondrial complex I impairment is central to the pathology of PD [26]. The results obtained in this study demonstrate that rotenone inhibited complex I function. More importantly, fucoidan (70 and 140 mg/kg) significantly relieved the inhibition of complex I function induced by rotenone. In contrast to the complex I, the complex II was not subjected to the damage by rotenone. This establishes that rotenone selectively inhibits the mitochondrial complex I, and such inhibition is attenuated by fucoidan. As readout, oxygen consumption of oxidations of site I and II substrates depends on not only the mitochondrial complexes I and II but also the distal complexes III and IV [27]. In eukaryotes, complexes I, III and IV form a supercomplex which is important for electron transport and proton pumps [28], while complexes II, III and IV contribute less energy to the overall electron transport. In this study, the oxidation of complex II-associated substrates remained unchanged, indicating that the pathway of complexes II, III and IV was most likely unaffected.

It is widely accepted that oxidative stress from the production of reactive oxygen species (ROS) is a principle contributor to the pathogenesis of PD. The mitochondrion is a major source of ROS and its dysfunction increases ROS formation. Excessive ROS can interact with cellular biomolecules especially DNA, lipids, and proteins. Increased oxidative products have been consistently observed in the SNpc of PD patients and PD animal models [29-31]. In this study, we also observed a significant increase in the oxidative products such as MDA, 3-NT, and 8-OHdG in the ventral midbrain of rotenone-lesioned PD rats. Encouragingly, fucoidan remarkably inhibited the increase of these oxidative products in accordance with previous reports [32]. These results suggest that fucoidan can enhance the mitochondrial function and thereby alleviate the oxidative stress in the ventral midbrain of rotenone-treated rats.

PGC-1α, as a transcriptional co-activator, plays a significant role in promoting mitochondrial biogenesis and enhancing their function [33-35]. A recent meta-analysis reported a decrease in PGC-1a and its downstream genes in postmortem brains of PD patients [36]. In PGC-1α knockout mice, expression of genes involved in mitochondrial respiration was markedly decreased and dopaminergic neurons were more sensitive to MPTP [37, 38]. In contrast, activation or overexpression of PGC-1a caused an increase in expression of nuclear-encoded subunits of the mitochondrial respiratory chain and prevented degeneration of dopaminergic neurons in cellular or animal models induced by mutant α-synuclein, rotenone, or MPTP [36, 38]. NRF2, a nuclear transcription factor, has been previously implicated in the activation of numerous nuclear genes that contribute to mitochondrial respiratory function [39]. The interaction of PGC-1α with NRF2 has been unequivocally confirmed by co-immunoprecipitation [40]. Through this interaction, PGC-1α enhanced mitochondrial capacity for oxidative phosphorylation and triggered the coordinate expression of nuclear-encoded genes driving mitochondrial biogenesis [41]. Therefore, the PGC-1α/NRF2 pathway is a promising target for pharmaceutical intervention of PD development [36]. In the present study, we found that rotenone decreased the expression of PGC-1α and NRF2 proteins in the ventral midbrain, while fucoidan significantly upregulated PGC-1α and NRF2 expression. Our results indicate that the upregulation of PGC-1α and NRF2 protein expression may contribute to the protective effect of fucoidan on mitochondrial function.

In summary, this study demonstrated, for the first time, that fucoidan alleviated PD-like behaviors and dopaminergic neurodegeneration in a rotenone-induced PD rat model. The neuroprotective effect of fucoidan may be partly mediated by enhancing mitochondrial respiratory function through the PGC-1α/NRF2 pathway. Future investigation using PGC-1α knockdown mice and other approaches would be useful for elucidating the molecular mechanisms of fucoidan-mediated mitochondria protection in the disease.
